# Temporomandibular condylar alterations in juvenile idiopathic arthritis most common in longitudinally severe disease despite medical treatment

**DOI:** 10.1186/1546-0096-12-43

**Published:** 2014-09-14

**Authors:** Anna-Lena Cedströmer, Margareta Ahlqwist, Anna Andlin-Sobocki, Lillemor Berntson, Britt Hedenberg-Magnusson, Lars Dahlström

**Affiliations:** Department of Behavioral and Community Dentistry, Institute of Odontology, Sahlgrenska Academy, University of Gothenburg, Göteborg, Sweden; Department of Oral and Maxillofacial Radiology, Institute of Odontology, Sahlgrenska Academy, University of Gothenburg, Göteborg, Sweden; Department of Orthodontics, Eastman Institutet, Folktandvården Stockholms län AB, Stockholm, Sweden; Department of Surgical Sciences, Oral and Maxillofacial Surgery, Uppsala University Hospital, Uppsala, Sweden; Department of Women’s and Children’s Health, Uppsala University, Uppsala, Sweden; Department of Dental Medicine, Section for Orofacial Pain and Jaw Function, Karolinska Institutet, Huddinge, Sweden; Department of Clinical Oral Physiology, Eastman Institutet, Folktandvården Stockholms län AB, Stockholm, Sweden

**Keywords:** Adolescent, Arthritis juvenile rheumatoid/diagnoses, Child, Retrospective studies, Temporomandibular joint

## Abstract

**Background:**

Juvenile idiopathic arthritis (JIA) is an autoimmune, heterogeneous disease and the temporomandibular joint (TMJ) can be affected, with consequences for mandibular growth and function. The aim of this study was to evaluate the importance of longitudinal medical treatment and the burden of disease activity on the development of temporomandibular condylar alterations as judged on panoramic radiographs.

**Methods:**

The study was a retrospective evaluation of dental and medical records in consecutive JIA patients referred to three specialist dental clinics in Sweden during an eight-year period. Data on the total pharmacological treatment and disease activity were evaluated longitudinally from disease onset to the time of the panoramic examination, during a median observation period of 2.5 years. The radiographs were analysed in terms of structural and shape alterations in the condyles and judged dichotomously.

**Results:**

Panoramic examinations were analysed in 158 patients from 266 referrals diagnosed with JIA. Condylar alterations (shape or structural) were seen in 68 patients (43%). Patients with condylar alterations were more extensively treated over time compared with those without condylar alterations. Powerful disease activity and/or potent medication at any time during the course of the disease implied an increased risk of alterations.

**Conclusions:**

Patients with JIA who require more intensive medication over time run the greatest risk of condylar alterations. As yet, current medical programmes have not been specified for the TMJ and more knowledge in this area is needed.

## Background

Juvenile idiopathic arthritis (JIA) is an autoimmune, heterogeneous disease with arthritis for at least six weeks and onset before the age of 16 [1]. The disease can express itself in many ways. The current classification was proposed in 1994 by the International League of Associations for Rheumatology (ILAR) [2, 3].

It has been suggested that the temporomandibular joint (TMJ) is one of the most frequently involved joints in JIA, according to radiographic changes [4]. Swelling is seldom seen and arthritis in the TMJ is often asymptomatic, as a result of which the TMJ has been called “the forgotten joint” in pediatric rheumatology [5]. The arthritis in the TMJ thus evolves without subjective or clinical expressions in many cases, which causes a delay in detection [6, 7], with consequences for mandibular growth and function [8]. A more precise clinical examination, combined with imaging of the TMJ, is therefore important for the detection of ongoing arthritis, as well as damage [9].

In the last few decades, magnetic resonance imaging (MRI) has become the new standard for examining the TMJs in children with JIA because it is an efficient method for detecting early inflammatory changes [10–12]. Computed tomography (CT) is also a widespread technique and is often used in this context [7, 13]. Arvidsson et al. [13] found that 70% of JIA patients had TMJ involvement on CT and MRI. MRI and CT are demanding, expensive examinations.

In contrast, panoramic radiography is simple, inexpensive, with relatively low radiation doses [14], commonly available and requires no sedation in young children. It is often performed prior to MRI or CT examinations in clinical practice. Previous studies have found condylar lesions on panoramic radiography in 17-78% of patients with JIA [15–19]. There is thus a substantial spread in results relating to the prevalence of condylar lesions among patients with JIA, even when the same radiological technique has been used.

The importance of current medication for TMJ arthritis in JIA has also been examined. Radiological TMJ abnormalities and the association with disease-modifying anti-rheumatic drugs (DMARD), such as methotrexate (MTX), are inconclusive. The results vary from no significant effect to reduced TMJ alterations in patients who medicate with MTX [17, 19, 20].

The degree of disease activity during its course could be expected to be of importance for the development of TMJ arthritis. Arvidsson et al. [19] found that TMJ abnormalities were associated with reduced well-being and more extensive involvement of other joints at the final examination 27 years after onset. Billau et al. [18], on the other hand, found no relationship between condylar lesions and disease activity after a median disease duration of 2.96 years.

The way pharmacological treatment and disease activity influence the development of TMJ arthritis longitudinally is unclear. The aim of this study was therefore to evaluate how longitudinal medical treatment and the burden of disease activity influence the development of temporomandibular condylar alterations as judged on panoramic radiographs.

## Methods

All consecutive patients who fulfilled the ILAR criteria for JIA [3] and were referred to three specialist dental clinics in Sweden during an eight-year period were considered. Another inclusion criterion was that at least one panoramic radiograph was performed during the study period. One dentist (A-LC) screened the dental records retrospectively and, supervised by a rheumatologist (LB), collected data from the medical records at the attending pediatric rheumatologic clinic.

Evaluations of the TMJs on the panoramic radiographs were made by one dentist (A-LC) and one specialist in oral radiology (MA) until consensus was reached. Both were blinded to all other dental and medical information. The latest performed panoramic examination was used for analysis. Seventy-two patients had only one panoramic radiograph performed.

**Figure 1:**
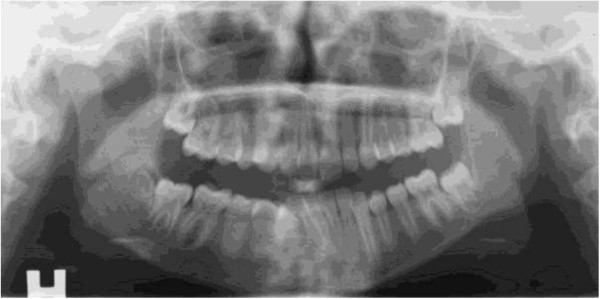
**Panoramic radiograph on a patient with JIA with example of common alteration, flattening, on the left TMJ.** Right side (H) is normal.

The panoramic radiographs were analysed in terms of structural and shape alterations in the condyles. Structural changes included erosion (area with diminished cortical density), sclerosis (increased cortical density) and subchondral cysts. Changes in the shape of the condyles included flattening (loss of smooth convexity) and osteophytes (bony process on the anterior condyle). A dichotomous judgement for each individual was given, alteration or not in one or both condyles. An example of common alterations is given in Figure 1. Moreover, any toadstool appearance as described by Petrovski et al. [21] in juvenile arthrosis and JIA was registered separately. In this condition the condyle is flattened, elongated and dorsally inclined. The condylar neck is shortened or absent.

If one or both condyles were judged as “unreadable”, the individual was excluded from further analysis. Most of the radiographs, 86%, were digital and analysed on monitors, one at the Department of Clinical Oral Physiology at the Eastman Institute in Stockholm and one at the Department of Oral and Maxillofacial Radiology, University of Gothenburg. Analogous radiographs, 14%, were analysed in an X-ray light box. Fifty randomly selected cases were evaluated until consensus was reached a second time after three weeks in order to establish the reliability of the evaluation over time.

The total pharmacological treatment was evaluated longitudinally from disease onset, established by the rheumatologist, to the time of the panoramic examination. Medication with DMARDs, which included MTX, and/or biologically modifying medications, including tumour necrosis factor alpha inhibitors (TNF-alpha) and/or corticosteroid injections in the TMJ, was denoted “potent medication”. Fifteen patients received corticosteroid injections in the TMJ but only 4 without simultaneous DMARDs and therefore further analyses were not possible. The use of “potent medication” for six months or more was denoted a “medication period”. An evaluation of medical treatment was performed for each six-month period during the first year after onset and thereafter once every year until panoramic examination. A period shorter than six months of medication or the use of non-steroidal anti-inflammatory drugs (NSAID) were not included.

Five disease activity groups yielding a modified version of the European League Against Rheumatism (EULAR) criteria [22] were used for the assessment of disease activity. Disease activity was thus recorded as active disease = EU 1 (increasing number of active joints), stable = EU 2 (unchanged number of active joints), inactive with treatment = EU 3, inactive without treatment = EU 4 (no disease activity and no treatment for less than two years) and remission = EU 5 (no disease activity and no treatment for more than two years). Disease activity, defined as EU 1 and/or 2 for six months or more, was denoted “active disease”. The first year after onset was evaluated for each six-month period during the first year and thereafter once every year. The time from disease onset to the date of the radiological examination could thus be described as the number of periods with “potent medication” and “active disease”. The number of “medication periods” and “active disease periods” varied from 0 to 8 and patients were followed for seven years at most.

### Statistics

In order to test the difference between condylar alterations or not, the Mantel-Haenszel chi-square test was used. For comparisons between condylar alterations or not in terms of gender and age, Fisher’s exact test and the Mann-Whitney u-test were used respectively. Spearman’s rank correlation was used for the correlation analysis. Two-tailed statistical analyses were performed at a significance level of 5%, unless otherwise stated. Kappa statistics were used to evaluate reliability over time. Bivariate logistic regression analysis was used to establish the risk of condylar alterations.

The multicentre study was performed with the approval of the Regional Ethical Review Board at the University of Gothenburg, Gothenburg, Sweden (Dnr 342-07).

## Results

A total of 266 patients with JIA were referred to the participating specialist dental clinics during the eight-year study period. Panoramic radiographs were exposed in 184 patients, 69%. Of the 184 patients, a total of 163 had medical data from the same year as the panoramic examination. Five radiographs were judged as “unreadable” and 158 patients were consequently included in this sub-study.

Five patients had missing medical data for one or two periods between onset and the panoramic examination.

The reliability of the dichotomous radiological assessments (structural and/or shape alteration or not) over time was kappa = 0.68 (95% CI 0.56 to 0.80). This strength of agreement is considered to be substantial [23].

The distribution of the 158 patients in ILAR categories, gender, age at onset, disease duration (time from onset, as judged by the rheumatologist, to the panoramic examination) radiographic condylar alterations (shape and/or structural) and toadstool appearances is shown in Table 1. Condylar alterations were found in 68 patients (43%) and were not significantly associated with category, gender, age at onset or duration. Sixty-seven patients had shape alterations and 25 structural alterations. The prevalence of toadstool appearances was 12. Eight of these also had condylar alterations.

**Table 1:** **JIA patients examined with panoramic radiographs (n = 158), allocated to the eight ILAR categories**

	Total	Systemic	Persistent oligo	Extended oligo	RF-negative polyarthritis	RF-positive polyarthritis	Psoriatic	ERA	Other arthritis
Total n (% girls)	158 (73)	4 (50)	62 (76)	12 (67)	43 (77)	10 (100)	17 (82)	2 (0)	8 (25)
Age at onset, median, 25/75th percentile yrs	7.2 (3.4/10.8)	4.8 (3.7/6.0)	6.0 (2.1/9.3)	4.4 (2.6/4.8)	7.1 (3.8/9.8)	11.7 (9.9/15.0)	9.0 (4.0/13.1)	13.5 (12.5/14.4)	10.1 (9.3/13.0)
Disease duration, median, 25/75th percentile yrs	2.5 (0.5/3.9)	2.9 (0.9/4.9)	2.6 (0.3/3.8)	2.4 (0.6/4.4)	3.0 (0.6/4.8)	0.9 (0.1/1.5)	2.6 (0.1/2.9)	0.3 (0.1/0.5)	1.7 (0.8/2.1)
Condylar alteration, n (%)	68 (43)	0	26 (42)	9 (69)	16 (37)	7 (70)	10 (56)	0	3 (33)
Toadstool appearance, n (%)	12 (8)	0	4 (6)	1 (8)	4 (9)	2 (20)	0	1	0

Distribution of age at onset, disease duration, condylar alteration (shape or structural) and toadstool appearance is given.

**Figure 2:**
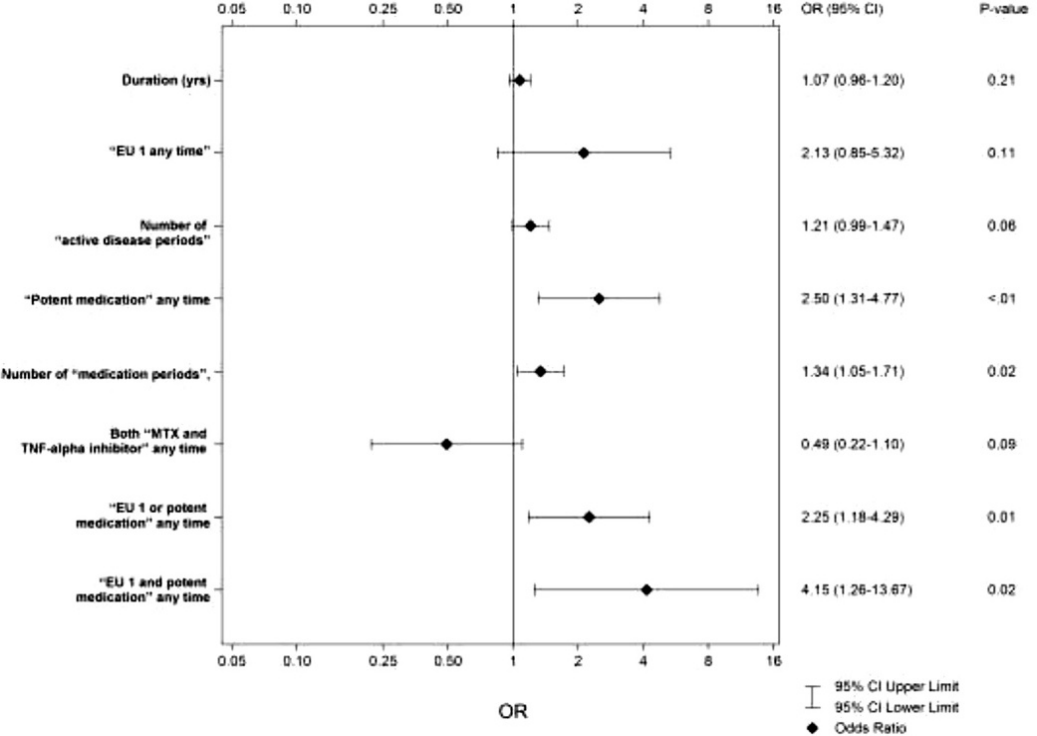
**Results of bivariate logistic regression on the risk of condylar alterations on panoramic radiographs in patients with JIA (n = 158) presented in odds ratios (OR) (95% Cl) and P-values.** The distribution of duration (time from onset to panoramic examination, yrs), “EU 1 any time” (active disease with increasing number of active joints any time for at least six months between onset and the panoramic examination), number of “active disease periods” (EU1 and/or EU2 (stable disease with unchanged number of active joints) for six months or more between onset and the panoramic examination), “potent medication” any time (MTX and/or TNF-alpha inhibitor and/or corticosteroid injections in the TMJ for at least six months between onset and the panoramic examination), number of “medication periods” (potent medication for six months or more between onset and the panoramic examination), “MTX + TNF-alpha inhibitor anytime” (intake of MTX and TNF-alpha any time for at least six months between onset and the panoramic examination). “EU 1 or potent medication” any time (EU 1 or potent medication for at least six months between onset and the panoramic examination) and “EU 1 and potent medication” any time (EU 1 and potent medication for at least six months between onset and the panoramic examination) are given.

Patients with or without condylar alterations (shape and/or structural) are compared in terms of length of active disease and burden of medication. The results are given in Table 2. Any “potent medication” and number of “medication periods” were more common in patients with condylar alterations. Moreover, “EU 1 or potent medication” and “EU 1 and potent medication” were significantly more common among patients with condylar alterations. The correlation between EU 1 and “potent medication” was r_s_ = 0.17 (P = 0.04).The results of the bivariate logistic regression are shown in the Figure 2. Patients with any “potent medication” ran a significantly increased risk of condylar alterations (P = 0.01). Increased numbers of “medication periods” also increased the risk of condylar alterations (P = 0.02). The risk of condylar alterations with a larger number of “active disease periods” was not statistically significant, only close to it (P = 0.06). Patients with “EU 1 or potent medication” ran an increased risk of condylar alterations (P = 0.01) and “EU 1 and potent medication” also increased this risk (P = 0.02).

**Table 2:** **A total of 158 patients with JIA examined with panoramic radiographs**

	Total	Condylar alterations	No condylar alterations	***P***
Total, n (% girls)	158 (73)	68 (78)	90 (70)	*0.26*
Duration, yrs, median (25/75th percentile)	2.5 (0.5/3.9)	2.8 (0.6/4.7)	2.3 (0.4/3.2)	*0.21*
“EU 1 any time”, n (%)	15 (9)	9 (13)	6 (7)	*0.11*
Number of “active disease periods”, median (25/75th percentile)	2.49 (1.0/3.0)	2.87 (1.0/4.0)	2.28 (1.0/3.0)	*0.06*
“Potent medication” any time, n (%)	75 (47)	41 (60)	34 (38)	***0.01***
Number of “medication periods”, median (25/75th percentile)	2.69 (1.0/4.0)	3.06 (1.0/4.0)	2.41 (1.0/3.0)	***0.02***
Both “MTX and TNF-alpha inhibitor” any time, n (%)	43 (27)	23 (34)	20 (22)	*0.09*
“EU 1 or potent medication” any time, n (%)	82 (52)	43 (63)	39 (43)	***0.01***
“EU 1 and potent medication” any time, n (%)	15 (10)	11 (16)	4 (4)	***0.02***

The association between condylar alterations, longitudinal disease activity and medication is presented. The bold values of column P is statistically significant (P =< 0.05).

Condylar alterations = shape or structural.

Duration = time from onset (yrs), as judged by the rheumatologist, to the panoramic examination.

“EU 1 any time” = active disease with increasing number of active joints any time for at least six months between onset and the panoramic examination.

Number of “active disease periods” = EU 1 and/or EU 2 (stable disease with unchanged number of active joints) for six months or more between onset and the panoramic examination.

“Potent medication” = MTX and/or TNF-alpha inhibitor and/or corticosteroid injections in the TMJ any time for at least six months between onset and the panoramic examination.

Number of “medication periods” = “potent medication” for six months or more between onset and the panoramic examination.

“MTX and TNF-alpha inhibitor any time” = intake of MTX and TNF-alpha any time for at least six months between onset and the panoramic examination.

“EU 1 and/or potent medication” any time = EU1 and/or potent medication for at least six months between onset and the panoramic examination.

## Discussion

This study collected data on TMJ condylar alterations related to longitudinal medical background variables in a comprehensive cohort of JIA patients referred to three specialist dental clinics in Sweden over an eight-year period. According to a National Care Program all JIA patients at rheumatologic clinics should be referred to specialist dental care but the percentage of JIA patients that is actually referred is unknown. The radiographically examined group comprised 69% of the total cohort of 266 patients. Condylar alterations, as judged on panoramic radiographs, were more common in patients with a heavier burden of longitudinal medication and consequently more severe illness.

The reasons for performing a panoramic examination in our study may have varied and it has previously been shown that patients with active disease, EU 2, were examined more often [24]. The fact that not all the patients were examined with panoramic radiography entails a possible bias. Missing medical data for solitary periods for some patients are also a shortcoming. Another weak point is that panoramic radiography has limited diagnostic value for TMJ. It provides an overview of the jaws, but only large condylar changes can be evaluated with any confidence compared with more advanced radiological techniques such as MRI, CT or Cone Beam Computed Tomography (CBCT) [25, 26]. The observational design also implies that the impact on the condyles in the event of no medication in active disease is unknown. A modified version of EULAR was used to describe disease activity in this study, but there are other ways of categorizing it. One limitation of EULAR is that the disease activity is only measured on active joints compared, for example, with JADAS 27, where the sums of components are compounded [27].

Condylar radiographic alterations were found in 43% of the examined patients. Studies using the panoramic radiographic technique have reported different results in terms of TMJ condylar alterations, 17-78%, and the size of the studied populations has also varied from 97 to 249 JIA patients [15–19]. Arvidsson et al. [19] reported that TMJ abnormalities in patients with JIA, found in panoramics, increased from childhood to adulthood from 42% to 82% in a cohort followed for 27 years.

Different scoring systems to grade the involvement of TMJs in JIA on panoramic examinations have been applied. Twilt et al. [9, 17] used a grading system developed for adult rheumatoid arthritis [28]. Billau et al. [18] used a “condylar damage score” from small cortical bone erosion to the complete absence of a condylar head. Pedersen et al. [16] graded condylar resorption from no radiological abnormalities to the total destruction of the condyle. Arvidsson et al. [19] graded the condylar deformity from 0 to 3. The divergent results for condylar alterations are most probably due to different scoring systems and patient populations. To simplify the categorization, condylar alterations were only registered dichotomously in this study.

The importance of longitudinal medication for avoiding TMJ arthritis in JIA, evaluated on panoramic radiographs, has previously been studied. Arvidsson et al. [19] found no significant difference in TMJ abnormalities between patients with or without previous MTX treatment and/or the use of biological drugs studied for a mean duration of 3.2 years. Twilt et al. [17] found TMJ involvement in 68% of the patients with previous or present DMARD (not MTX) as compared to 29% in patients with previous or present immunosuppressive therapy (corticosteroids and MTX) with a mean duration of 4.9 years. The difference between the groups was not discussed in more detail. Ince et al. [20], on the other hand, found significantly more frequent and more severe condylar involvement in JIA patients without MTX at the time of the panoramic examination. The observation period was eight years for the non-MTX group and 6.9 years for the MTX group. It was concluded that MTX therapy may minimise the TMJ destruction, but longitudinal studies would provide more definitive information.

As yet, there is not enough knowledge when it comes to the optimal therapy for avoiding or treating TMJ arthritis. TNF-alpha inhibitors are used in approximately 20-25% of children with JIA in Sweden [29]. A review concluded that some children with JIA develop TMJ arthritis despite TNF-alpha inhibitors [4]. The authors suggested that there is a need for more longitudinal studies to evaluate the effect on the TMJs. A high percentage of children with JIA may have TMJ arthritis whether or not they are on systemic DMARD therapy. [30]. Studies comparing TNF-alpha with MTX treatment have not been performed [31]. In this cohort, some patients had TNF-alpha inhibitors for only a short period and it is therefore difficult to draw any conclusions about the effect on TMJ arthritis. Although not statistically significant in this study the combination of TNF-alpha inhibitors and MTX may have a protective effect on the TMJ. Corticosteroid injections in the TMJ might be effective [32] but this therapy alone was very uncommon in this cohort.

In a review of randomized controlled trials in JIA on treatment with biological agents, no conclusions about efficacy could be drawn, because of the small number of patients and differences in design between the trials [33]. Many children with JIA have periods of active disease despite current medical programmes. Vidqvist et al. [34] reported several inflamed joints in the last year in 58% of patients with JIA with a median age of 19 years in spite of medication. The present study confirms their result. The number of “active disease periods” correlated with periods of heavier medical treatment and, in our study, periods of heavier treatment were significantly associated with condylar alterations.

## Conclusion

To summarize, TMJ damage, evaluated as condylar alterations on panoramic radiographs, was fairly common in our cohort of patients with JIA. Alterations have been found to correlate with synovitis [35]. The unique anatomy of the TMJ with a thin layer of fibrocartilage at the surface of the condylar head and the biochemical composition makes it susceptible to damage caused by arthritis [4]. The results suggest, within the limits of this observational study, that active disease appears to increase the risk of alterations to the TMJs despite medication. As yet, current medical programmes have not been specified for the TMJ and more knowledge is needed in this area. Longer follow-up studies and further prospective studies with the emphasis on the progress of TMJ arthritis are necessary.
